# Molecular evolution of the crustacean hyperglycemic hormone family in ecdysozoans

**DOI:** 10.1186/1471-2148-10-62

**Published:** 2010-02-25

**Authors:** Nicolas Montagné, Yves Desdevises, Daniel Soyez, Jean-Yves Toullec

**Affiliations:** 1 UPMC Univ Paris 06, UMR A 1272 INRA - Physiologie de l'Insecte: Signalisation et Communication, F-75005, Paris, France; 2 UPMC Univ Paris 06, FRE 3247 CNRS - Modèles en Biologie Cellulaire et Évolutive, Observatoire Océanologique, F-66651, Banyuls-sur-Mer, France; 3 UPMC Univ Paris 06, ER3 - Biogenèse des Signaux Peptidiques, F-75005, Paris, France; 4 UPMC Univ Paris 06, UMR 7144 CNRS - Adaptation et Diversité en Milieu Marin, Station Biologique de Roscoff, F-29682, Roscoff, France

## Abstract

**Background:**

Crustacean Hyperglycemic Hormone (CHH) family peptides are neurohormones known to regulate several important functions in decapod crustaceans such as ionic and energetic metabolism, molting and reproduction. The structural conservation of these peptides, together with the variety of functions they display, led us to investigate their evolutionary history. CHH family peptides exist in insects (Ion Transport Peptides) and may be present in all ecdysozoans as well. In order to extend the evolutionary study to the entire family, CHH family peptides were thus searched in taxa outside decapods, where they have been, to date, poorly investigated.

**Results:**

CHH family peptides were characterized by molecular cloning in a branchiopod crustacean, *Daphnia magna*, and in a collembolan, *Folsomia candida*. Genes encoding such peptides were also rebuilt *in silico *from genomic sequences of another branchiopod, a chelicerate and two nematodes. These sequences were included in updated datasets to build phylogenies of the CHH family in pancrustaceans. These phylogenies suggest that peptides found in Branchiopoda and Collembola are more closely related to insect ITPs than to crustacean CHHs. Datasets were also used to support a phylogenetic hypothesis about pancrustacean relationships, which, in addition to gene structures, allowed us to propose two evolutionary scenarios of this multigenic family in ecdysozoans.

**Conclusions:**

Evolutionary scenarios suggest that CHH family genes of ecdysozoans originate from an ancestral two-exon gene, and genes of arthropods from a three-exon one. In malacostracans, the evolution of the CHH family has involved several duplication, insertion or deletion events, leading to neuropeptides with a wide variety of functions, as observed in decapods. This family could thus constitute a promising model to investigate the links between gene duplications and functional divergence.

## Background

The study of the evolutionary history of arthropods is a challenging field of research, as they constitute the large majority of known metazoan species and exhibit an extreme diversity of body plans and physiology. Nowadays, evo-devo approaches largely contribute to this research area [reviewed in [1]], but the evolution of peptide hormone families directly involved in physiological adaptations remains poorly investigated in arthropods, especially when compared to vertebrates. The Crustacean Hyperglycemic Hormone (CHH) family could constitute a model of choice to address this question, as CHH family peptides are well known in decapod crustaceans, where they do play major roles in many physiological processes. Since decapods were the first invertebrates where a neuroendocrine mechanism was discovered, they have been extensively used in comparative endocrinology studies, and CHH has become the archetype of a neuropeptide family including around 150 members to date [reviewed in [2-4]]. CHH family peptides are 72 to 78 amino acids long and are, based on structural features, divided into two sub-families [5]. Type I peptides, the CHHs *sensu stricto*, are pleiotropic hormones involved in the regulation of energetic and ionic metabolism and, in addition, can also exert an inhibitory effect on molting and reproduction [reviewed in [6]]. By a tissue-specific alternative splicing mechanism, CHH genes produce a second peptide, devoid of hyperglycemic activity, named CHH-L (for CHH long isoform), which may be involved in osmoregulation [7-9]. Type II peptides, namely the molt-inhibiting hormones (MIHs), the vitellogenesis-inhibiting hormones (VIHs) and the mandibular organ-inhibiting hormones (MOIHs), are functionally more specialized than CHHs. Characterized through their inhibitory actions on molting and reproduction, they never elicit hyperglycemia [reviewed in [2,10]].

**Figure 1:**
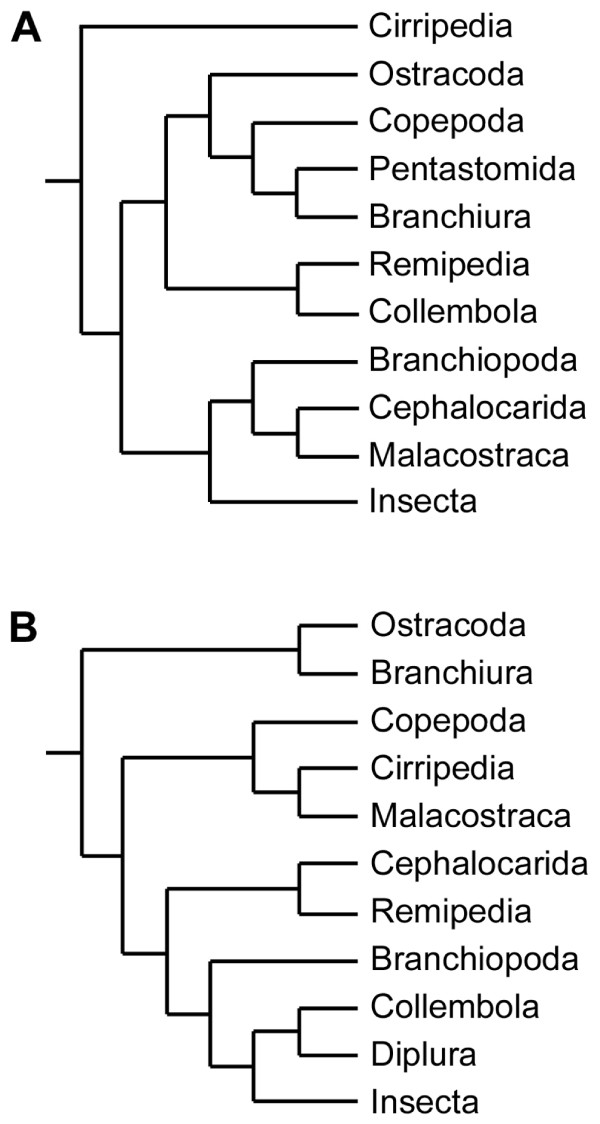
**Current hypotheses about the relationships of pancrustacean taxa**. (A) Phylogeny built from 12 mitochondrial protein-encoding genes [19]. (B) Phylogeny built from the analysis of nuclear protein-encoding genes *EF-1α, EF-2 *and *POLII *[23].

The evolution of CHHs has been recently discussed in decapods [11] and we have intended here to extend the evolutionary study to the entire family, by including data from non-decapod taxa. Little is known about the CHH family outside decapods, but it may be present in the entire Arthropoda, and even in all Ecdysozoa. Indeed, a CHH and a VIH were isolated from an isopod crustacean [12,13], and neuropeptides sharing the same structural signature, the ion transport peptides (ITPs), were also characterized in several insect species [reviewed in [14]]. As for decapod CHHs, ITP genes produce an ITP-L isoform by alternative splicing, whose function remains elusive. To go further, heterologous immunostainings revealed the occurrence of CHH-like peptides in two branchiopods [15], a myriapod [16] and a chelicerate [17], and a gene was found in the genome of the nematode *Caenorhabditis elegans*, which encodes a putative peptide exhibiting a CHH family structural signature [3]. To increase our knowledge on the CHH family in the taxa cited above, we have conducted an *in silico *study on available genomic sequences from arthropods and nematodes. Exon-intron patterns of the genes were determined and sequences of putative CHH family peptides were deciphered. In addition, CHH family sequences were obtained by molecular cloning in a collembolan and in a branchiopod, whose phylogenetic positions are currently uncertain. Indeed, the grouping of crustaceans and hexapods in a Pancrustacea clade is widely accepted but the relationships inside Pancrustacea remain controversial. Recent molecular phylogenies based on mitochondrial genes (Figure 1A) notably yield a paraphyletic Hexapoda, with Collembola separated from Insecta, and Branchiopoda as a sister-group to Malacostraca and Cephalocarida [18-20]. These phylogenies largely differ from those based on nuclear genes (Figure 1B), where Hexapoda appears monophyletic, and Branchiopoda are generally sister-group to Hexapods [21-23].

Newly characterized sequences were included in CHH family sequence datasets, which were then used to build phylogenies of this family and to investigate its evolutionary history. Because of the reduced size of these datasets, a test was conducted to assess which of the two phylogenetic hypotheses presented Figure 1 was more strongly supported by our data, and could be further used as a basis to elaborate evolutionary scenarios of the CHH family genes.

## Results

### CHH family peptides characterized in *Daphnia magna *and *Folsomia candida*

**Figure 2:**
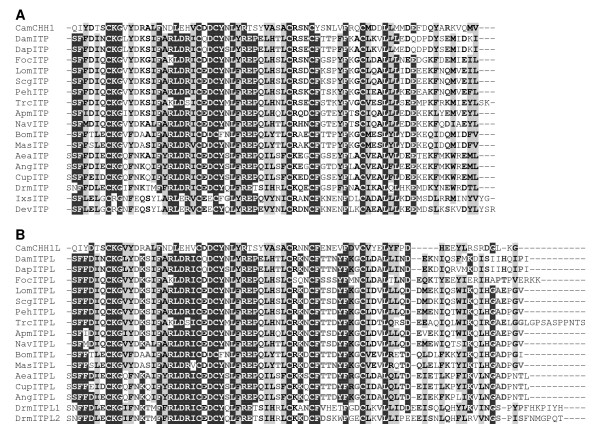
**Alignments of the putative ITP (ion transport peptide) and ITP-L sequences from branchiopods, collembolans, insects and chelicerates**. (A) Multiple-sequence alignment of 17 ITP sequences with CHH (crustacean hyperglycemic hormone) sequence from the shore crab *Carcinus maenas*. (B) Multiple-sequence alignment of 16 ITP-L sequences with CHH-L sequence from *Carcinus maenas*. Aea: *Aedes aegypti *; Ang: *Anopheles gambiae *; Apm: *Apis mellifera *; Bom: *Bombyx mori *; Cam: *Carcinus maenas *; Cup: *Culex pipiens *; Dam: *Daphnia magna *; Dap: *Daphnia pulex *; Dev: *Dermacentor variabilis *; Drm: *Drosophila melanogaster *; Foc: *Folsomia candida *; Ixs: *Ixodes scapularis *; Lom: *Locusta migratoria *; Mas: *Manduca sexta *; Nav: *Nasonia vitripennis *; Peh: *Pediculus humanus *; Scg: *Schistocerca gregaria *; Trc: *Tribolium castaneum*. Sequence accession numbers are given in Table 2.

In the branchiopod *Daphnia magna*, RT-PCRs conducted with degenerate primers designed from insect ITP transcripts and followed by 3'-5'RACE produced two complete cDNAs. Their length was 1053 and 1198 bp, respectively, and they encoded peptide precursors composed of a signal peptide, a precursor-related peptide and a mature hormone. The two cDNA sequences were identical except for a 145 bp stretch (position 383 to 527) present in only one of them, suggesting the occurrence of an alternative splicing during mRNA processing. The short cDNA encoded a 72-residue peptide with a C-terminus end putatively amidated, which was named ion transport peptide (ITP) as it appeared to be related to insect ITPs (Figure 2A). On the other hand, the long cDNA encoded a non-amidated peptide of 79 amino acids, only differing from ITP after the fortieth residue, which was named ITP-L (Figure 2B).

The two complete cDNA sequences obtained from the collembolan *Folsomia candida *led to similar results: two transcripts of different lengths (1307 and 2018 bp) encoded prepro-peptides which only differed after the fortieth residue of the mature peptide. The 72 residue amidated peptide, translated from the short cDNA, was named ITP and the 82 residue non-amidated peptide, translated from the longest cDNA, was named ITP-L (Figure 2A and 2B).

### Sequences characterized in *silico*

For the insects *Anopheles gambiae, Apis mellifera, Culex pipiens, Nasonia vitripennis *and *Pediculus humanus*, nucleotide sequences encoding for ITP or ITP-L, which have not been recovered by automatic annotation programs were deduced from the gene sequence following alternative splicing rules. A gene encoding putative ITP and ITP-L isoforms was also found in the genome of another water flea, *Daphnia pulex*. At the present time, both ITP (72 or 73 residue amidated peptides) and ITP-L (79 to 90 residue non-amidated peptides) sequences are available from 12 insect and two branchiopod species (Figure 2A and 2B).

In the genome of the deer tick *Ixodes scapularis *(Chelicerata), a gene was found that encoded only one mature ITP-like peptide. In addition, cDNA and peptide sequences of another ITP-like peptide were found, during database mining, in the American dog tick *Dermacentor variabilis*. These peptides are 74 and 75 residue long, respectively, and are not amidated on their C-terminal end (Figure 2A).

**Figure 3:**
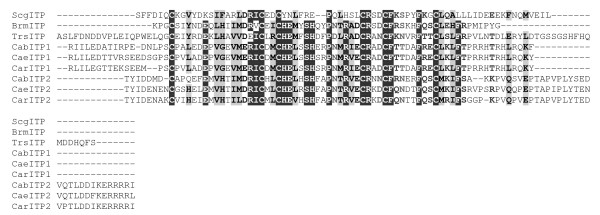
**Alignment of putative CHH family peptides of nematodes**. ITP-like sequences deduced from nematode genomes were aligned with the ITP sequence of *Schistocerca gregaria*. Brm: *Brugia malayi *; Cab: *Caenorhabditis briggsae *; Cae: *Caenorhabditis elegans *; Car: *Caenorhabditis remanei *; Scg: *Schistocerca gregaria *; Trs: *Trichinella spiralis*.

In the *Caenorhabditis elegans *genome, the gene *ZC168.2 *has been described as putatively encoding a CHH family peptide of 83 amino acid residues [3]. In the present study, a second gene (*C05E11.6*) which may encode another putative CHH family peptide was found in the *C. elegans *genome: this gene encodes a mature peptide of 97 amino acids containing six cysteyl residues whose positions are similar to those found in CHH family hormones (Figure 3). The corresponding peptides ZC168.2 and C05E11.6 were named ITP 1 and ITP 2, respectively, based on their similarity with insect ITPs and without predicting any physiological function. Similar ITP 1 and ITP 2 peptides were also deduced from the genomes of *Caenorhabditis briggsae *and *Caenorhabditis remanei*. Finally, single CHH family peptides were deduced from the genomes of the Malaysian filarial worm *Brugia malayi *and the whipworm *Trichinella spiralis*. These putative peptides were 102 and 60 amino acids long, respectively (Figure 3).

**Figure 4:**
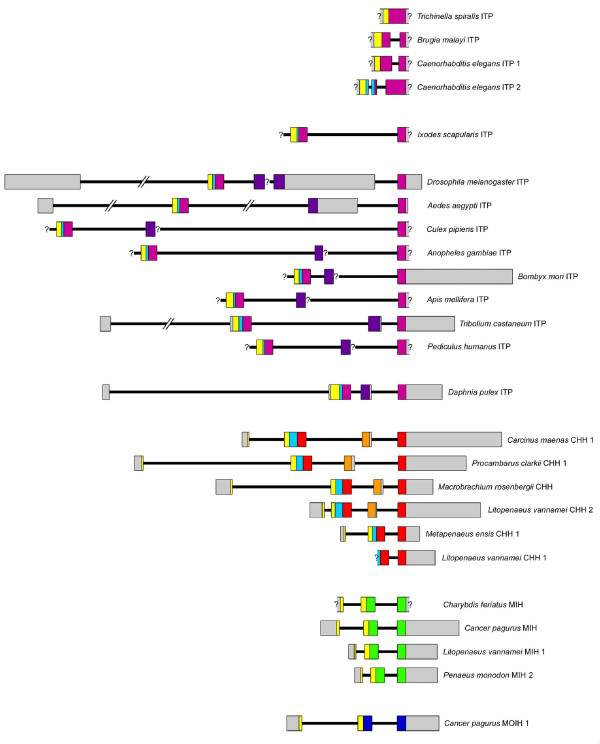
**Structure of the genes encoding CHH family peptides**. Exons are represented by open boxes: untranslated regions are in grey, regions encoding signal peptides are in yellow, those encoding precursor-related peptides in light blue, those encoding mature ITPs, CHHs, MIHs and MOIHs in purple, red, green and dark blue, respectively, and alternatively spliced exons encoding ITP-L and CHH-L C-terminus regions in violet and orange, respectively. Question marks indicate untranslated regions whose precise borders are not known.

For each of the genes described here, exon-intron structures were determined and compared to those already described (Figure 4). These data were further used, in addition to phylogenetic information, to infer evolutionary scenarios of the CHH family.

### Phylogeny of CHH family peptides in Pancrustacea

**Figure 5:**
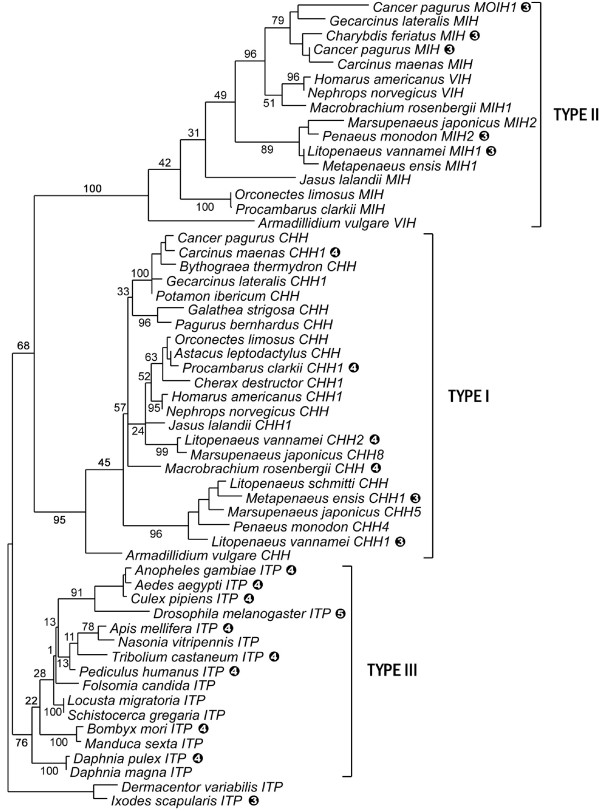
**Phylogeny of the CHH family in Pancrustacea, based on maximum likelihood analysis of an amino acid dataset (56 taxa, 73 characters)**. The analysis was carried out using a WAG+I+G model of protein evolution. *Ixodes scapularis *and *Dermacentor variabilis *putative ITPs were assigned as outgroup. Numbers at nodes are bootstrap values based on 100 replicates. Sequence accession numbers are given in Table 2. For taxa in which the genes have been sequenced, the number of exons (three, four or five) is indicated in a black circle after the name of the species. A four-exon pattern was assigned for taxa in which two peptides arising by alternative splicing have been described

In the maximum-likelihood (ML) phylogeny of pancrustacean CHH family peptides (Figure 5), type I and type II peptides of decapods were both arranged in monophyletic sub-groups. Type I and II peptide clades were also found in the phylogeny built by Bayesian inference (BI) based on the DNA dataset. The "type I" clade, supported by a bootstrap value of 95% in the ML analysis (posterior probability, or pp, of 1.00 in BI), contained all CHHs from decapods, with the CHH from the isopod *Armadillidium vulgare *at their base. Similarly, the "type II" clade, supported by a bootstrap value of 100% (pp of 1.00 in BI), contained all MIHs, VIHs and MOIHs from decapods, with the isopod VIH at their base. This topology suggests that the type I and type II peptide genes constitute paralogous lineages, which have appeared by duplication of an ancestral gene before the radiation of malacostracans (taxa that contains decapods and isopods). Inside the type I clade, the clade of Dendrobranchiata (peneid shrimps) CHHs encoded by three-exon genes is at the base of the other decapod CHHs, encoded by four-exon genes, found both in Dendrobranchiata and Pleocyemata (the remaining decapods). It suggests that a duplication of a CHH gene has occurred before the radiation of decapods and the split between Dendrobranchiata and Pleocyemata, but this node was strongly supported only in the BI analysis (pp of 1.00, bootstrap value of 45% in the ML analysis).

A clade supported by a bootstrap value of 76% (pp of 0.99 in BI) was positioned at the base of the type I and type II clades and contained all ITPs from insects, together with the peptides characterized in the collembolan *Folsomia candida *and the branchiopods *Daphnia magna *and *Daphnia pulex*, which were located at the base. The type III sub-family was thus created to designate peptides contained in this clade.

Since the topology of this clade was only weakly supported (notably with insects appearing paraphyletic), amino acid and DNA datasets containing less taxa but more characters were used for phylogenetic inference. When an outgroup (DNA or amino acid sequences from *Ixodes scapularis*) was included in these datasets, a structure of two monophyletic groups was found within the ingroup: a "type I" clade (supported by a bootstrap value of 100% in the ML analysis based on the amino acid dataset and by posterior probabilities of 1.00 in BI analyses using either amino acid or DNA dataset) and a "type III" clade (bootstrap value of 48% in the ML analysis of the amino acid dataset, posterior probabilities of 0.79 and 0.98 in BI analyses based on the amino acid and the DNA datasets, respectively). Since the four exons used in these datasets exist only in the ingroup species, the inclusion of the outgroup resulted in a loss of phylogenetic signal. Then, the outgroup was removed for subsequent analyses and the resulting trees were rooted using the structure in two clades previously found.

**Figure 6:**
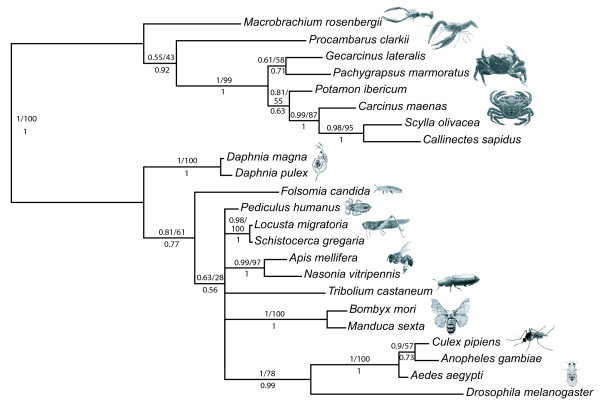
**Phylogeny based on Bayesian analysis of the amino acid dataset including CHH-L and ITP-L sequences (23 taxa, 109 characters)**. Trees obtained by maximum likelihood analysis of the amino acid dataset and by Bayesian analysis of the DNA dataset were fully congruent. Numbers above branches are posterior probabilities and bootstrap values (based on 100 replicates) obtained from the analysis of the amino acid dataset, and numbers below branches are posterior probabilities obtained from the analysis of the DNA dataset. Sequence accession numbers are given in Table 2.

Phylogenies built from these 23-taxa datasets using ML and BI methods were similar (Figure 6). Insects appeared monophyletic, albeit with moderate support, and the relationships within the insect clade were not resolved. Yet, the collembolan *Folsomia candida *was positioned at the base of the insects, thus forming a moderately supported monophyletic hexapod clade, and branchiopods were a sister group to hexapods, with high support.

### Evolutionary scenarios of the CHH family genes

**Figure 7:**
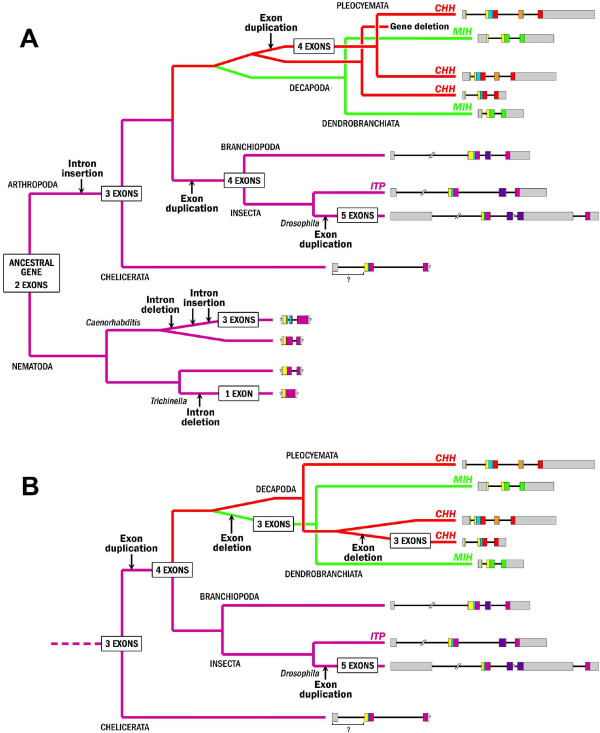
**Evolutionary scenarios of the CHH family genes in Ecdysozoa**. (A) Hypothesis accounting for two independent exon duplications originating the four-exon genes, which may have occurred on one side in a common ancestor of decapods and on the other side in a common ancestor of insects and branchiopods. (B) Hypothesis accounting for a single exon duplication originating the four-exon genes of insects, branchiopods and decapods.

Using the 23-taxa sequence datasets, a KH test was conducted to decide which of the two competing phylogenetic hypotheses for arthropods presented in Figure 1 could be used to build an evolutionary scenario of the CHH family. This test clearly supported (P < 0.0001) the tree based on nuclear genes, with a monophyletic Hexapoda sister-group to Branchiopoda (Figure 1B), versus the tree based on mitochondrial genes (Figure 1A). Then, the ML phylogeny of CHH family peptides (Figure 5) was superimposed on a species tree in which Branchiopoda were sister-group to Hexapoda, to create a topology accounting for gene duplications in Pancrustacea (Figure 7A). In nematodes, a gene duplication was also inferred in the *Caenorhabditis *phylum, as two paralogs were found in genomes of *Caenorhabditis *species. In the most parsimonious scenario of gene structure evolution based on this topology, CHH family genes of Ecdysozoa evolved from an ancestral two-exon gene, with nine partial insertion or deletion events (Figure 7A). In this scenario, CHH family genes of arthropods have all evolved from a three-exon gene, and the four-exon gene structure would have appeared independently twice, in a common ancestor of decapods and in a common ancestor of insects and branchiopods.

Because of the weak support, in the ML phylogeny (Figure 5), at the node indicating the duplication which led to the two paralogous CHH genes found in Dendrobranchiata (with three or four exons), an alternative topology was considered for pancrustacean CHH family genes, where the CHH gene duplication would have occurred only in the Dendrobranchiata phylum (Figure 7B). In that case, the most parsimonious scenario implied eight intron or exon insertion/deletion events, and the exon duplication leading to the four-exon gene structure would have occurred only once.

Regardless of the evolutionary scenario considered, the putative ancestral gene was likely composed of two exons and a phase 2 intron interrupting the codon of the amino acid residue following the fourth cysteine of the mature peptide. Such an intron was found in every known CHH family gene, except the ITP2 genes in *Caenorhabditis *species and the ITP gene of *Trichinella spiralis *(Figure 4).

Dibasic processing sites used for precursor maturation by prohormone convertase then likely appeared independently twice, in the common ancestor to arthropods on one hand and in the common ancestor to the *Caenorhabditis *genus on the other hand, leading to the occurrence in these lineages of precursor related peptides (blue boxes in Figures 4 and 7), such as the CHH-precursor related peptides (CPRPs) of decapods.

## Discussion

### Evolution of the CHH family in decapods and functional implications

Since the discovery of neuroendocrine factors within the eyestalks of decapods seventy years ago [24-26], structural and functional data about CHH family peptides in these crustaceans have been accumulated. While our knowledge increased, it became more and more difficult to propose a model of endocrine regulation applying to all decapods. Indeed, CHH family peptides are not identical in all decapod groups, and their function varies with the species. This diversity prompted us to investigate the evolutionary history of these peptides.

In decapods, many gene duplications have occurred, leading to two main paralogous lineages (type I and type II peptides) and to a large polymorphism of CHH family peptides inside these lineages (Figure 7). For example, two clusters containing each at least 7 different CHH genes were identified in the shrimp *Metapenaeus ensis *[27]. A paradigm in evolutionary biology is that gene duplication represents the major mechanism for emergence of new functions, as one of the two copies is potentially freed from selective pressure and will accumulate more mutations during evolution [28,29]. In the CHH family, the functional divergence observed between the two paralogous lineages could result from a subfunctionalization [30,31], rather than from a neofunctionalization. In fact, the ancestral gene may have encoded a pleiotropic hormone, like current CHHs, and after duplication of this gene the type II paralogous lineage may have evolved faster to peptides with a CHH-like structure, but devoid of hyperglycemic activity and conversely exhibiting more specialized activities, such as inhibition of molting or vitellogenesis (for MIHs and VIHs, respectively). The faster evolution rate of type II peptide genes may explain the loss of phylogenetic signal observed in MIHs, VIHs and MOIHs sequences. Indeed, the topology of the "type II" clade (Figure 5) is not congruent with the phylogeny of decapods, whereas it is for the "type I" clade, as shown in earlier work [11]. This clearly impedes the elucidation of evolutionary relationships between type II peptides. The only paralogous lineages clearly identified so far are the MIHs and MOIHs of *Cancer *crabs, as the MOIH genes seem to have appeared from a duplicated MIH gene only in this genus [32]. In lobsters, there is no MIH identified so far but a VIH instead, whose gene may have evolved from a MIH gene subjected to a reduced selective pressure, as molt inhibition is exerted in this taxa by a CHH isoform [33].

### Evolutionary history "outside decapods"

A high number of gene duplications were observed in decapods only. So far, the only paralogous lineages identified outside decapods are the "ITP1" and "ITP2" genes found in *Caenorhabditis *species (Figure 7), and only one CHH family peptide gene could be found in genomes of the other nematodes, the insects and the deer tick. In the water flea *Daphnia pulex*, one ITP gene was also rebuilt from whole genome shotgun (WGS) sequences but other WGS sequences sharing sequence identity with the ITP gene have been found. However, it is unclear whether these sequences correspond to another CHH family peptide coding gene or to a pseudo-gene: this gene would have a peculiar structure, with exon III lacking, and the prepro-peptide putatively encoded would lack the dibasic processing site found in every CHH or ITP precursors and the 6 following residues of the mature peptide as well. Yet, although only one ITP and one ITP-L cDNA have been cloned in *Daphnia magna*, recently published EST data suggest that both genes may be expressed in *Daphnia pulex *[34], but EST and genomic sequences do not match (only 85% identity between deduced amino acid sequences). To include these data in an evolutionary scenario, genomic and mRNA sequences should be characterized by molecular cloning.

This apparent lack of paralogous lineages outside decapods raises the question of a possible conservation of function between the ITPs of insects and the related peptides found in branchiopods, chelicerates and nematodes. Yet, functional studies are too scarce to address this question. In insects, the effect of ITP on ionic and water balance was only demonstrated in locusts [35,36] and nothing is known about the function of ITP-like peptides in chelicerates or nematodes. In branchiopods, only one gene expression study on sex determination in *Daphnia magna *has indicated that CHH family peptides may inhibit methyl farnesoate synthesis by the mandibular organ [37]. It is noteworthy that several CHH family peptides are known to play such a role in decapods [38-40]. It will be informative to determine whether *Daphnia *peptides exhibit other typical "decapod" functions such as hyperglycemia or ecdysteroid synthesis inhibition, or if they also possess an anti-diuretic activity, as shown for insect ITPs. A similar question about another neuropeptide family, the RPCH/AKH family, has recently found a partial answer. Like for the CHH family, the putative RPCH (red pigment-concentrating hormone) of *Daphnia magna *shares more sequence similarity with AKHs (adipo-kinetic hormones) of insects than with RPCH of decapod crustaceans [41]. In a heterologous bioassay, this peptide was able to trigger a mobilization of lipid reserves when injected into the green shield bug *Nezara viridula *whereas it did not provoke pigment migration when injected into the shrimp *Palaemon pacificus *[42]. Although heterologous bioassays must be considered cautiously, such results suggest that this peptide could have an adipokinetic function in water fleas (like in insects) rather than a pigment-concentration function (like in decapods).

### Emergence of the four-exon genes

One important step in the evolution of the CHH family is the emergence of the four-exon genes found in decapods, branchiopods and insects. In these genes, exons III and IV encode peptide sequences of similar lengths, with two cystein residues in the same positions, thus suggesting that the supplementary exon has appeared by tandem duplication of another exon. At first glance, it seems more likely that this duplication has occurred only once, in a common ancestor of Pancrustacea (Figure 7B). In all these taxa, two peptides are produced by alternative splicing; one peptide (CHH or ITP) is 72 or 73 amino acids long and is always amidated on its C-terminal end, and the other peptide (CHH-L or ITP-L) is slightly longer and is never amidated. Moreover, the expression of these two isoforms seems to be similar in decapods and insects; in the crab *Carcinus maenas *and the caridean shrimp *Macrobrachium rosenbergii*, CHH transcripts were mainly found in neurons of the central nervous system (more precisely in the X-organ located in the eyestalk) whereas CHH-L transcripts were found in neurons of the peripheral nervous system [7,8]; in the moths *Manduca sexta *and *Bombyx mori *and the locust *Schistocerca americana*, ITP-immunoreactive neurons were only found in the brain, whereas ITP-L-immunoreactive ones were found along the ventral nervous chain. ITP-L immunoreactivity was also detected in the brain, but was weak and restricted to cell bodies, thus suggesting that ITP-L was not secreted from this tissue [43].

Yet, even if the hypothesis of an independent emergence of the fourth exon (Figure 7A) appears unlikely, it should not be rejected, as a similar exon duplication also occurred in a *Drosophila *ancestor, leading to the five-exon gene found in *Drosophila melanogaster *[44].

### Controversy about pancrustacean phylogeny

Given the relative short length of the sequences used, the objective of this study was not to build a phylogeny of pancrustaceans using CHH family peptides, but to choose the appropriate phylogeny to infer evolutionary scenarios of the CHH family. The KH test carried out with our datasets indicated a congruence of the data with recent molecular phylogenies of pancrustaceans based on nuclear gene sequences [21-23] and not with those based on mitochondrial genes [18-20]. Moreover, the robustness and quality of the signal contained in mitochondrial gene sequences for arthropod phylogenetics have recently been questioned [45,46]. Supplementary taxa will have to be represented within CHH family datasets to confirm this result, but studying the evolutionary history of this family may help to select synapomorphies to support concurrent phylogenetic hypotheses about pancrustacean relationships. For example, the presence of paralogous type I and type II peptides could constitute a trait shared by malacostracans and their sister-groups.

## Conclusion

The evolutionary scenarios proposed in this study suggest that genes encoding Crustacean Hyperglycemic Hormone family peptides of Ecdysozoa evolved from an ancestral two-exon gene. In the malacostracan lineage (including decapods), the evolution of the CHH family has involved numerous duplications, insertions and deletions of exons or entire genes, leading to the wide variety of functions displayed by the encoded neurohormones in decapods. This neuropeptide family could thus constitute a promising model to investigate the evolutionary forces at the root of the functional divergence of duplicated genes. Outside malacostracans, the number of gene duplications seems to have been lower, which may reflect different evolutionary pathways. At present, CHH family peptides characterized outside decapods are still scarce, and this work constitutes a first step in a wider quest. In the future, the evolutionary scenarios elaborated here will be completed and amended, as CHH family peptides will probably be characterized in new taxa. The 38 genome sequencing projects currently under way in ecdysozoan species will be of valuable help as soon as they are completed, to enlarge our view on the evolution of this multigenic family.

## Methods

### Biological material and RNA extraction

*Daphnia magna *and *Folsomia candida *(strain TO) specimens were supplied by the BIOEMCO laboratory and the Ecology & Evolution laboratory, respectively, both located at the Ecole Normale Supérieure (Paris, France). Total RNA was extracted from 10 mg of whole animals, using SV Total RNA Isolation System (Promega, Madison, WI). cDNA was synthesized from 300 ng of total RNA using 200 U of M-MLV Reverse Transcriptase (Promega), 20 pmoles of each dNTP and 30 pmoles of an Oligo(dT) primer. This synthesis was performed at 42°C for 1 hour, followed by an inactivation step at 70°C for 15 min.

### PCR amplification and cloning

First, a central region of putative ITP precursor cDNAs was amplified using degenerate primers DgnITP-Up (5'-CTTCGAYATCMAKTGYAARGG-3') and DgnITP-Down (5'-RAGRCAWCCTTTGAAGWATG-3') designed from the alignment of known insect ITP cDNAs. The reaction mixture contained 1.5 U of GoTaq^® ^DNA Polymerase (Promega), 5 pmoles of each dNTP and 10 pmoles of each degenerate primer. Amplifications were conducted with a denaturation step at 94°C for 3 min, followed by 40 cycles of 94°C for 30 sec, 55°C for 30 sec and 72°C for 30 sec, and a final extension step at 72°C for 10 min. After their purification with the Wizard^® ^SV Gel and PCR Clean-Up System (Promega), products were cloned using pGEM^®^-T Easy Vector and JM109 Competent Cells (Promega). Plasmids were purified using the Wizard^® ^*Plus *SV Minipreps DNA Purification System (Promega), and their sequencing was performed by Cogenics - Genome Express (Meylan, France).

### 3' and 5'RACE

To create cDNAs which contain a synthetic adaptor either at the 5' or 3' end, total RNA was reverse-transcribed using the SMART™ RACE cDNA Amplification Kit (Clontech, Moutain View, CA). For PCR amplifications, two sets of primers, specific of *Daphnia magna *and *Folsomia candida*, were designed from the partial cDNA sequences obtained by RT-PCR. These primers are listed in Table 1. First amplifications were performed using the adaptor primer supplied with the kit (Universal Primer Mix) and specific primers Up1 (for 3'RACE) or Down1 (for 5'RACE). Then, nested PCRs were performed on 1/150^th ^of the first PCR products, between the Nested Universal Primer from the kit and specific primers Up2 or Down2. All reverse-transcription and PCR amplification steps were carried out following the protocol supplied in the kit. RACE products were cloned and sequenced as described above.

**Table 1:** Primers used for 3' and 5'RACE.

Primer	Species	Sequence (5' to 3')	Tm (°C)
DamITP-Up1	*Daphnia magna*	TCCAGCGGCCATCATTCGTTGTCC	71
DamITP-Up2	*Daphnia magna*	CCTCGACCGCATTTGCCAAGACTG	71
DamITP-Down1	*Daphnia magna*	GGACAACGAATGATGGCCGCTGGA	71
DamITP-Down2	*Daphnia magna*	ACTGGGAAGCACTGCGAGCAAAACC	72
FocITP-Up1	*Folsomia candida*	GCCAAGCTGGACAGAATTTGCGACGAT	73
FocITP-Up2	*Folsomia candida*	ACAACTCCACTCGCTATGCAGGTCGAA	73
FocITP-Down1	*Folsomia candida*	TTCGACCTGCATAGCGAGTGGAGTTGT	73
FocITP-Down2	*Folsomia candida*	ATCGTCGCAAATTCTGTCCAGCTTGGC	73

### Database mining

A search for CHH family peptide sequences from arthropods and nematodes was performed with the BLAST program at the NCBI website http://blast.ncbi.nlm.nih.gov/Blast.cgi. The non-redundant protein sequences (nr) database was mined by blastp algorithm using *Schistocerca gregaria *ITP or the putative peptide ZC168.2 of *Caenorhabditis elegans *as query sequences for arthropods and for nematodes, respectively. Corresponding mRNA sequences were also collected, and aligned with genome sequences to determine the exon-intron structure of the genes. Sequences of genes encoding putative CHH family peptides of *Daphnia pulex *(Branchiopoda), *Ixodes scapularis *(Chelicerata), *Caenorhabditis remanei *and *Brugia malayi *(Nematoda) were assembled from whole genome shotgun (WGS) sequences available in the NCBI Trace Archives database, by assembling trace reads with CLC Genomics Workbench software (CLCbio, Aarhus, Denmark).

### Phylogenetic analyses

Datasets containing mature CHH family peptide sequences and the corresponding DNA sequences (when available) from Pancrustacea were created. In the amino acid dataset, 37 sequences from decapods (22 CHH and 15 MIH, VIH or MOIH) were selected among the 90 available sequences, and added to the 17 sequences from hexapods and branchiopods. ITP-like sequences of the ticks *Ixodes scapularis *and *Dermacentor variabilis *were included to be used as outgroup (for sequence references, see Table 2). Alignments were performed manually with Se-Al v2.0a11 http://tree.bio.ed.ac.uk/software/seal/, and after removal of N-terminal and C-terminal unconserved residues, the dataset contained 56 taxa and 73 characters. The DNA dataset contained 49 taxa and 219 characters.

**Table 2:** Amino acid sequences used for phylogenetic inference.

Species	Peptide	Accession number
**Chelicerata**		
**Arachnida**		
*Dermacentor variabilis*	ITP	ACC99599
*Ixodes scapularis*	ITP	^(1)^
		
**Hexapoda**		
**Collembola**		
*Folsomia candida*	ITP	ACF15252
	ITP-L	ACJ01668
		
**Insecta**		
*Aedes aegypti*	ITP	AAY29661
	ITP-L	AAY29663
*Anopheles gambiae*	ITP	^(1)^
	ITP-L	XP_313928
*Apis mellifera*	ITP	XP_001120062
	ITP-L	^(1)^
*Bombyx mori*	ITP	AAY29659
	ITP-L	AAY29660
*Culex pipiens*	ITP	^(1)^
	ITP-L	XP_001845654
*Drosophila melanogaster*	ITP	ABZ88142
	ITP-L 2	ABZ88141
*Locusta migratoria*	ITP	AAD20820
	ITP-L	AAD20821
*Manduca sexta*	ITP	AAY29657
	ITP-L	AAY29658
*Nasonia vitripennis*	ITP	^(1)^
	ITP-L	XP_001604056
*Pediculus humanus*	ITP	^(1)^
	ITP-L	EEB14555
*Schistocerca gregaria*	ITP	AAB16822
	ITP-L	AAB16823
*Tribolium castaneum*	ITP	ABN79658
	ITP-L	ABN79657
		
**Branchiopoda**		
*Daphnia magna*	ITP	ABO43963
	ITP-L	ABO43964
*Daphnia pulex*	ITP	^(1)^
	ITP-L	^(1)^
		
**Malacostraca**		
**Isopoda**		
*Armadillidium vulgare*	CHH	P30814
	VIH	P83627
		
**Decapoda**		
*Astacus leptodactylus*	CHH	AAX09331
*Bythograea thermydron*	CHH	AAK28329
*Callinectes sapidus*	CHH	AAS45136
	CHH-L	ABC61678
*Cancer pagurus*	CHH	P81032
	MIH	CAC05346
	MOIH 1	CAB61424
*Carcinus maenas*	CHH 1	AAG29429
	CHH-L 1	AAG29432
	MIH	Q27225
*Charybdis feriatus*	MIH	O96605
*Cherax destructor*	CHH 1	P83485
*Galathea strigosa*	CHH	ABS01332
*Gecarcinus lateralis*	CHH 1	ABF48652
	CHH-L	ABF58091
	MIH	ABF06632
*Homarus americanus*	CHH 1	P19806
	VIH	P55320
*Jasus lalandii*	CHH 1	P56687
	MIH	P83220
*Litopenaeus schmitti*	CHH	P59685
*Litopenaeus vannamei*	CHH 1	Q26181
	CHH 2	[56]
	MIH 1	ABD73291
*Macrobrachium rosenbergii*	CHH	AAL40915
	CHH-L	AAL40916
	MIH 1	AAL37948
*Marsupenaeus japonicus*	CHH 5	O15981
	CHH 8	^(1)^
	MIH2	BAD36757
*Metapenaeus ensis*	CHH 1	AAD11813
	MIH 1	O76534
*Nephrops norvegicus*	CHH	AAQ22391
	VIH	AAK58133
*Orconectes limosus*	CHH	Q25589
	MIH	P83636
*Pachygrapsus marmoratus*	CHH	AAO27804
	CHH-L	AAO27806
*Pagurus bernhardus*	CHH	ABE02191
*Penaeus monodon*	CHH 4	O97386
	MIH 2	AAR89517
*Potamon ibericum*	CHH	ABA70560
	CHH-L	ABA70561
*Procambarus bouvieri*	CHH 2	Q10987
*Procambarus clarkii*	CHH 1	BAA89003
	CHH-L 1	AAL79193
	MIH	P55848
*Scylla olivacea*	CHH	AAQ75760
	CHH-L	ABP88270

^**(1) **^Sequences deduced from genomic sequences or from the alternatively spliced product

DNA and amino acid datasets including CHH-L and ITP-L sequences were also created. For the 23 taxa in which such peptides are known, the amino acid sequence of the mature CHH-L or ITP-L (encoded by exons II and III of the gene) was concatenated with the C-terminal sequence of CHH or ITP (encoded by exon IV of the gene). Alignments were performed manually and 109 unambiguously aligned residues were conserved in the dataset. The DNA dataset contained 327 characters.

At first, DNA and translated amino acid sequences of *Ixodes scapularis *ITP-like were included in these datasets, to be used as an outgroup in order to determine the global branching order in the ingroup. Since the gene encoding this peptide lacks exon III in the outgroup taxon, corresponding characters were replaced with "?" symbols in the alignments. Because of the loss of phylogenetic signal brought by the inclusion of the outgroup, it was removed from datasets for subsequent phylogenetic analyses, but the global structure previously found was kept.

Phylogenetic reconstructions were carried out using Bayesian inference and maximum likelihood. Bayesian analyses were performed with MrBayes 3.1.2 with four chains of 10^6 ^generations, trees sampled every 100 generations and burn-in value set to 20% of the sampled trees. We checked that standard deviation of the split frequencies fell below 0.01 to insure convergence in tree search. Protein sequences were analyzed with a mixed amino acid model [47], and DNA sequences were considered with an evolutionary model designed for coding sequences and taking the genetic code into account [48-50].

Maximum likelihood reconstructions were carried out only on amino acid sequences. For both datasets, the WAG+I+G substitution model [51] was determined as the best-fit model of protein evolution by ProtTest 1.3 [52]http://darwin.uvigo.es/software/prottest_server.html, following Akaike Information Criterion. Rate heterogeneity was set at four categories. The gamma distribution parameter and the proportion of invariable sites were estimated from the datasets. Tree reconstructions were performed using PhyML 3.0 [53]http://www.atgc-montpellier.fr/phyml/ and validated with 100 bootstrap replicates.

### Evolutionary scenarios

The two phylogenetic trees shown in Figure 1 were used as competing hypotheses to assess if one was more strongly supported by the 23-taxa sequence datasets. This was done via a KH test using a maximum likelihood criterion [54]. This allowed us to choose a "species tree" on which the "gene tree" could be super-imposed.

The evolution of CHH family gene structures was analyzed using Mesquite 2.6 [55], under a parsimony framework. For this analysis, two alternate topologies depicting the evolution of the CHH family genes were created. All the known intron locations within CHH family genes were considered as characters and the presence/absence of intron in each location was coded 0 (absence) or 1 (presence) to create a character matrix.

## Authors' contributions

NM carried out molecular cloning, database mining, sequence alignments and drafted the manuscript. YD performed phylogenetic analyses and edited the manuscript. DS participated in the design of the study and edited the manuscript. JYT designed and coordinated the study and helped to draft the manuscript. All authors read and approved the final manuscript.

## References

[B1] Angelini DR Kaufman TC Comparative developmental genetics and the evolution of arthropod body plans Annu Rev Genet 2005 39 95 119 10.1146/annurev.genet.39.073003.112310 16285854

[B2] Böcking D Dircksen H Keller R Wiese K The crustacean neuropeptides of the CHH/MIH/GIH family: structures and biological activities The Crustacean Nervous System 2002 Berlin, Heidelberg, New York: Springer 84 97

[B3] Chen SH Lin CY Kuo CM In silico analysis of crustacean hyperglycemic hormone family Mar Biotechnol (NY) 2005 7 3 193 206 10.1007/s10126-004-0020-5 15933902

[B4] Soyez D Fingerman M, Nagabhushanam R Recent data on the crustacean hyperglycemic hormone family Recent Advances in Marine Biotechnology 2003 10 Plymouth, U.K.: Science Publishers 279 301

[B5] Lacombe C Grève P Martin G Overview on the sub-grouping of the crustacean hyperglycemic hormone family Neuropeptides 1999 33 1 71 80 10.1054/npep.1999.0016 10657474

[B6] Fanjul-Moles ML Biochemical and functional aspects of crustacean hyperglycemic hormone in decapod crustaceans: review and update Comp Biochem Physiol C Toxicol Pharmacol 2006 142 3-4 390 400 10.1016/j.cbpc.2005.11.021 16403679

[B7] Chen SH Lin CY Kuo CM Cloning of two crustacean hyperglycemic hormone isoforms in freshwater giant prawn (*Macrobrachium rosenbergii*): evidence of alternative splicing Mar Biotechnol (NY) 2004 6 1 83 94 10.1007/s10126-003-0014-8 14583813

[B8] Dircksen H Böcking D Heyn U Mandel C Chung JS Baggerman G Verhaert P Daufeldt S Plosch T Jaros PP Crustacean hyperglycaemic hormone (CHH)-like peptides and CHH-precursor-related peptides from pericardial organ neurosecretory cells in the shore crab, *Carcinus maenas*, are putatively spliced and modified products of multiple genes Biochem J 2001 356 1 159 170 10.1042/0264-6021:3560159 11336648 PMC1221824

[B9] Tiu SH He JG Chan SM The LvCHH-ITP gene of the shrimp (*Litopenaeus vannamei*) produces a widely expressed putative ion transport peptide (LvITP) for osmo-regulation Gene 2007 396 2 226 235 10.1016/j.gene.2007.02.027 17466469

[B10] Webster SG Coast GM, Webster SG Neuropeptides inhibiting growth and reproduction in crustaceans Recent Advances in Arthropod Endocrinology 1998 Cambridge, U.K.: Cambridge University Press 33 52

[B11] Montagné N Soyez D Gallois D Ollivaux C Toullec JY New insights into evolution of crustacean hyperglycaemic hormone in decapods - first characterization in Anomura FEBS J 2008 275 5 1039 1052 10.1111/j.1742-4658.2007.06245.x 18298796

[B12] Grève P Sorokine O Berges T Lacombe C Van Dorsselaer A Martin G Isolation and amino acid sequence of a peptide with vitellogenesis inhibiting activity from the terrestrial isopod *Armadillidium vulgare *(Crustacea) Gen Comp Endocrinol 1999 115 3 406 414 10.1006/gcen.1999.7330 10480992

[B13] Martin G Sorokine O Van Dorsselaer A Isolation and molecular characterization of a hyperglycemic neuropeptide from the sinus gland of the terrestrial isopod *Armadillidium vulgare *(Crustacea) Eur J Biochem 1993 211 3 601 607 10.1111/j.1432-1033.1993.tb17587.x 8436119

[B14] Dircksen H Insect ion transport peptides are derived from alternatively spliced genes and differentially expressed in the central and peripheral nervous system J Exp Biol 2009 212 3 401 412 10.1242/jeb.026112 19151215

[B15] Zhang Q Keller R Dircksen H Crustacean hyperglycaemic hormone in the nervous system of the primitive crustacean species *Daphnia magna *and *Artemia salina *(Crustacea: Branchiopoda) Cell Tiss Res 1997 287 3 565 576 10.1007/s004410050779 9023085

[B16] Laverdure AM Carette-Desmoucelles C Breuzet M Descamps M Neuropeptides and related nucleic acid sequences detected in peneid shrimps by immunohistochemistry and molecular hybridizations Neuroscience 1994 60 2 569 579 10.1016/0306-4522(94)90265-8 8072696

[B17] Stockmann R Laverdure AM Breuzet M Localization of a crustacean hyperglycemic hormone-like immunoreactivity in the neuroendocrine system of *Euscorpius carpathicus *(L.) (Scorpionida, Chactidae) Gen Comp Endocrinol 1997 106 3 320 326 10.1006/gcen.1997.6874 9204365

[B18] Carapelli A Liò P Nardi F Wath E van der Frati F Phylogenetic analysis of mitochondrial protein coding genes confirms the reciprocal paraphyly of Hexapoda and Crustacea BMC Evol Biol 2007 7 suppl2 S8 2 13 17767736 10.1186/1471-2148-7-S2-S8 PMC1963475

[B19] Cook CE Yue Q Akam M Mitochondrial genomes suggest that hexapods and crustaceans are mutually paraphyletic Proc R Soc B 2005 272 1295 1304 10.1098/rspb.2004.3042 16024395 PMC1564108

[B20] Hassanin A Phylogeny of Arthropoda inferred from mitochondrial sequences: strategies for limiting the misleading effects of multiple changes in pattern and rates of substitution Mol Phylogenet Evol 2006 38 100 106 10.1016/j.ympev.2005.09.012 16290034

[B21] Mallatt J Giribet G Further use of nearly complete 28S and 18S rRNA genes to classify Ecdysozoa: 37 more arthropods and a kinorhynch Mol Phylogenet Evol 2006 40 772 794 10.1016/j.ympev.2006.04.021 16781168

[B22] Timmermans MJ Roelofs D Marien J van Straalen NM Revealing pancrustacean relationships: phylogenetic analysis of ribosomal protein genes places Collembola (springtails) in a monophyletic Hexapoda and reinforces the discrepancy between mitochondrial and nuclear DNA markers BMC Evol Biol 2008 8 83 10.1186/1471-2148-8-83 18366624 PMC2315649

[B23] Regier JC Shultz JW Kambic RE Pancrustacean phylogeny: hexapods are terrestrial crustaceans and maxillopods are not monophyletic Proc R Soc B 2005 272 395 401 10.1098/rspb.2004.2917 15734694 PMC1634985

[B24] Abramowitz RK Abramowitz AA Moulting, growth, and survival after eyestalk removal in *Uca pugilator* Biol Bull 1940 78 179 188 10.2307/1537771

[B25] Brown FA Cunningham O Influence of the sinusgland of crustaceans on normal viability and ecdysis Biol Bull 1939 77 104 114 10.2307/1537849

[B26] Smith RI Studies on the effects of eyestalk removal upon young crayfish (*Cambarus clarkii*, Girard) Biol Bull 1940 79 145 152 10.2307/1537835

[B27] Gu PL Yu KL Chan SM Molecular characterization of an additional shrimp hyperglycemic hormone: cDNA cloning, gene organization, expression and biological assay of recombinant protein FEBS Lett 2000 472 122 128 10.1016/S0014-5793(00)01420-4 10781818

[B28] Ohno S Evolution by Gene Duplication Berlin, Heidelberg 1970 New York: Springer-Verlag

[B29] Zhang J Evolution by gene duplication: an update Trends Ecol Evol 2003 18 6 292 298 10.1016/S0169-5347(03)00033-8

[B30] Hughes AL The evolution of functionally novel proteins after gene duplication Proc R Soc B 1994 256 1346 119 124 10.1098/rspb.1994.0058 8029240

[B31] Lynch M Force A The probability of duplicate gene preservation by subfunctionalization Genetics 2000 154 1 459 473 10629003 10.1093/genetics/154.1.459 PMC1460895

[B32] Lu W Wainwright G Webster SG Rees HH Turner PC Clustering of mandibular organ-inhibiting hormone and moult-inhibiting hormone genes in the crab, *Cancer pagurus*, and implications for regulation of expression Gene 2000 253 2 197 207 10.1016/S0378-1119(00)00282-1 10940557

[B33] Chang ES Bruce MJ Newcomb RW Purification and amino acid composition of a peptide with molt-inhibiting activity from the lobster *Homarus americanus* Gen Comp Endocrinol 1987 65 56 64 10.1016/0016-6480(87)90222-X 3803902

[B34] Gard AL Lenz PH Shaw JR Christie AE Identification of putative peptide paracrines/hormones in the water flea *Daphnia pulex *(Crustacea; Branchiopoda; Cladocera) using transcriptomics and immunohistochemistry Gen Comp Endocrinol 2009 160 3 271 287 10.1016/j.ygcen.2008.12.014 19135444

[B35] King DS Meredith J Wang YJ Phillips JE Biological actions of synthetic locust ion transport peptide (ITP) Insect Biochem Mol Biol 1999 29 11 18 10.1016/S0965-1748(98)00098-8 10070740

[B36] Phillips JE Meredith J Audsley N Richardson N Macins A Ring M Locust ion transport peptide (ITP): A putative hormone controlling water and ionic balance in terrestrial insects Amer Zool 1998 38 3 461 470

[B37] Eads BD Andrews J Colbourne JK Ecological genomics in *Daphnia*: stress responses and environmental sex determination Heredity 2008 100 2 184 190 10.1038/sj.hdy.6800999 17519967

[B38] Keller R Kegel G Reichwein B Sedlmeier D Soyez D Roubos EW, Wendelaar Bonga SE, Vaudry H, De Loof A Biological effects of neurohormones of the CHH/MIH/GIH peptide family in crustaceans Recent Developments in Comparative Endocrinology and Neurobiology 1999 Nijmegen: Shaker 209 212

[B39] Laufer H Johnson M Demir N Twidy K Chang E Soyez D Van Herp F Bagshaw J Identification of lobster hyperglycemic hormones (CHHs) with mandibular organ inhibiting activity SICB Annual Meeting: 2003; Toronto, Canada 2003

[B40] Wainwright G Webster SG Wilkinson MC Chung JS Rees HH Structure and significance of mandibular organ-inhibiting hormone in the crab, *Cancer pagurus *- Involvement in multihormonal regulation of growth and reproduction J Biol Chem 1996 271 22 12749 12754 10.1074/jbc.271.22.12749 8662685

[B41] Christie AE Cashman CR Brennan HR Ma M Sousa GL Li L Stemmler EA Dickinson PS Identification of putative crustacean neuropeptides using *in silico *analyses of publicly accessible expressed sequence tags Gen Comp Endocrinol 2008 156 2 246 264 10.1016/j.ygcen.2008.01.018 18321503

[B42] Marco HG Gäde G Biological activity of the predicted red pigment-concentrating hormone of *Daphnia pulex *in a crustacean and an insect Gen Comp Endocrinol 2010 166 1 104 110 10.1016/j.ygcen.2009.08.002 19686751

[B43] Dai L Zitnan D Adams ME Strategic expression of ion transport peptide gene products in central and peripheral neurons of insects J Comp Neurol 2007 500 353 367 10.1002/cne.21192 17111378

[B44] Dircksen H Tesfai LK Albus C Nassel DR Ion transport peptide splice forms in central and peripheral neurons throughout postembryogenesis of *Drosophila melanogaster* J Comp Neurol 2008 509 1 23 41 10.1002/cne.21715 18418898

[B45] Cameron SL Miller KB D'Haese CA Whiting MF Barker SC Mitochondrial genome data alone are not enough to unambiguously resolve the relationships of Entognatha, Insecta and Crustacea *sensu lato *(Arthropoda) Cladistics 2004 20 534 557 10.1111/j.1096-0031.2004.00040.x 34892962

[B46] Delsuc F Phillips MJ Penny D Comment on "Hexapod origins: monophyletic or paraphyletic?" Science 2003 301 1482 10.1126/science.1086558 12970547

[B47] Ronquist F Huelsenbeck JP MrBayes 3: Bayesian phylogenetic inference under mixed models Bioinformatics 2003 19 1572 1574 10.1093/bioinformatics/btg180 12912839

[B48] Goldman N Yang Z A codon-based model of nucleotide substitution for protein-coding DNA sequences Mol Biol Evol 1994 11 725 736 7968486 10.1093/oxfordjournals.molbev.a040153

[B49] Muse SV Gaut BS A likelihood approach for comparing synonymous and nonsynonymous nucleotide substitution rates, with application to the chloroplast genome Mol Biol Evol 1994 11 715 724 7968485 10.1093/oxfordjournals.molbev.a040152

[B50] Shapiro B Rambaut A Drummond AJ Choosing appropriate substitution models for the phylogenetic analysis of protein-coding sequences Mol Biol Evol 2006 23 1 7 9 10.1093/molbev/msj021 16177232

[B51] Whelan S Goldman N A general empirical model of protein evolution derived from multiple protein families using a maximum-likelihood approach Mol Biol Evol 2001 18 5 691 699 11319253 10.1093/oxfordjournals.molbev.a003851

[B52] Abascal F Zardoya R Posada D ProtTest: Selection of best-fit models of protein evolution Bioinformatics 2005 21 2104 2105 10.1093/bioinformatics/bti263 15647292

[B53] Guindon S Gascuel O A simple, fast and accurate method to estimate large phylogenies by maximum-likelihood Syst Biol 2003 52 5 696 704 10.1080/10635150390235520 14530136

[B54] Kishino H Hasegawa M Evaluation of the maximum likelihood estimate of the evolutionary tree topologies from DNA sequence data, and the branching order in hominoidea J Mol Evol 1989 29 2 170 179 10.1007/BF02100115 2509717

[B55] Maddison WP Maddison DR Mesquite: a modular system for evolutionary analysis 2009 http://mesquiteproject.org Version 2.619199524

[B56] Tsutsui N Ohira T Kawazoe I Takahashi A Wilder MN Purification of sinus gland peptides having vitellogenesis-inhibiting activity from the whiteleg shrimp *Litopenaeus vannamei* Mar Biotechnol (NY) 2007 9 3 360 369 10.1007/s10126-006-6151-0 17357858

